# Effect of Traditional Chinese Medicine Injection on Cancer-Related Fatigue: A Meta-Analysis Based on Existing Evidence

**DOI:** 10.1155/2020/2456873

**Published:** 2020-12-21

**Authors:** Zhengkai Huang, Qiang Zhang, Yihua Fan, Jiajing Zhou, Mingkun Liang, Xin Deng, Jian Liang

**Affiliations:** ^1^College of Integrated Traditional Chinese and Western Medicine, Hunan University of Chinese Medicine, Changsha 410208, China; ^2^Department of Oncology, Army Medical Center of PLA, ChongQing 400042, China; ^3^First Teaching Hospital of Tianjin University of Traditional Chinese Medicine, Tianjin 300193, China; ^4^National Clinical Research Center for Chinese Medicine Acupuncture and Moxibustion, Tianjin 300381, China; ^5^Department of Oncology, Yantai Hospital of Traditional Chinese Medicine, Yantai 264000, China; ^6^Department of Science and Technology, Ruikang Hospital Affiliated to Guangxi University of Chinese Medicine, Nanning 530011, China; ^7^School of Basic Sciences, Guangxi University of Chinese Medicine, Nanning 530200, China; ^8^College of Medical, Guangxi University, Nanning 530004, China

## Abstract

**Methods:**

We systematically searched randomized controlled studies reported through March 2020 in PubMed, EMBASE, the Cochrane Central Register of Controlled Trials, Web of Science, China Biomedical Literature Database (CBM), the China National Knowledge Infrastructure (CNKI), Wanfang, and VIP databases. Two investigators independently screened the studies according to the predetermined criteria, extracted data, and evaluated the bias risk of the included studies, using RevMan5.3 software.

**Results:**

Twelve studies enrolling 1005 participants were included in this systematic review. We found that TCMJ could improve the clinical efficacy of CRF patients (RR = 1.24, 95% CI: 1.05–1.46, *P*=0.01), ameliorate fatigue status (RR = 1.44, 95% CI: 1.27–1.65, *P* < 0.00001), and improve quality of life (MD = 8.34, 95% CI: 3.31–13.37, *P*=0.001), but there was no statistical significance in the fatigue score (MD = −1.10, 95% CI: −2.23–0.04, *P*=0.06). Referring to the number of adverse events, the safety of TCMJ was good. Subgroup analysis showed that TCMJ could improve clinical efficacy, fatigue, and quality of life in a short time (≤4 weeks). Among them, tonic TCMJ could improve the clinical efficacy. TCMJ had advantages in improving fatigue of lung cancer and gastric cancer. In addition, life quality of lung cancer patients improved significantly.

**Conclusion:**

Current research evidence showed that TCMJ could improve the clinical efficacy, fatigue status, and life quality of patients with CRF. In addition, we found that TCMJ could improve the clinical efficacy of CRF patients in a short period of time. Tonic TCMJ could improve the clinical efficacy, but heat-clearing TCMJ could not. Life quality and fatigue status of lung cancer patients improved significantly. However, due to the sample size and quality of the included studies, the results of this analysis should be treated with caution. The above conclusions still need to be verified by more large-sample and high-quality randomized controlled trials.

## 1. Introduction

The National Comprehensive Cancer Network (NCCN) defines cancer-related fatigue (CRF) as a persistent, subjective fatigue associated with cancer or cancer treatment, which is out of proportion to recent activity and interferes with normal function [1]. In addition, among cancer survivors, about 1/4 to 1/3 of patients developed CRF for 10 consecutive years after the cancer was diagnosed [2]. CRF is a common and painful side effect associated with chemotherapy, radiotherapy, and the tumor itself [3–5]. Chemotherapy has considerable toxicity and is an important factor for the fatigue of cancer patients [6]. 50% to 90% of patients feel tired [7–9]. The physical, emotional, and mental health of CRF patients are affected, reducing their quality of life and potentially reducing the overall survival rate of patients [10, 11]. Although a large number of pharmacological and clinical studies have been carried out, CRF has not been fully treated due to the limited understanding of its pathophysiology [12, 13]. Today, traditional Chinese medicine injection (TCMJ) is widely used in adjuvant treatment of cancer, delaying cancer progression, strengthening the immune system, and improving complications and side effects caused by chemotherapy [14, 15]. TCMJ for CRF mainly includes Kangai injection, Aidi injection, Shenmai injection, and Shenqi Fuzheng injection. In the traditional Chinese medicine theory, CRF belongs to the category of “consumptive disease”. Due to the consumptive internal injuries and the deficiency of viscera function, phlegm, dampness, blood stasis, and other pathogenic factors are endogenous. The chemotherapeutic drugs, which are heat-toxic, consume gas and injure blood, and affect the functions of the spleen, liver, and kidney [16]. Studies have found that TCMJ can inhibit the production of proinflammatory cytokines by peripheral immune cells and enhance antitumor immunity through PDL1, TIM3, FOXP3, and other targets, so as to improve fatigue symptoms [17]. Clinical studies have shown that TCMJ is superior to conventional treatment in improving fatigue symptoms in 113 patients with CRF [18].

In recent years, the efficacy of TCMJ for CRF patients has been gradually confirmed by related studies. In order to further evaluate the efficacy and safety of TCMJ in the treatment of CRF patients, randomized controlled trials (RCTs) were studied by a meta-analysis method. We evaluated the quality of RCT strictly according to the evaluation principles of evidence-based medicine, extracted data, and meta-analyzed RCTs in accordance with the inclusion criteria, with a view to evaluating the clinical efficacy of TCMJ in CRF treatment and providing evidence for clinical treatment and study.

## 2. Materials and Methods

This review was conducted in accordance with the Preferred Reporting Items for Systematic Reviews and Meta-Analyses (PRISMA) guidelines and the recommendations of the Cochrane Collaboration.

### 2.1. Literature Screening and Identification of Relevant Studies

We searched PubMed, EMBASE, the Cochrane Library, Web of Science, China Biomedical Literature Database (CBM), China National Knowledge Infrastructure, Wanfang, and VIP databases up to March 2020. The search terms included “cancer related fatigue,” “CRF,” “injection,” and “Traditional Chinese medicine”. We reviewed all retrieved articles by reading the titles and abstracts. Then, the full text of the possibly relevant studies was examined for further suitability evaluations in our present meta-analysis. Meanwhile, to identify more eligible studies, we searched the references of related studies manually. The whole studies in the meta-analysis were firstly published in the primary literature with no reproduction in other studies.

### 2.2. Inclusion Criteria

The inclusion criteria were as follows: (1) population: patients with cancer-related fatigue, clear diagnosis, and unlimited cancer types, regardless of age and gender; (2) intervention: the experimental group was treated with both control group treatment and TCMJ, and the type of TCMJ was not limited; (3) comparison: the control group was treated with chemotherapy alone; (4) study design: RCT, language limited to English and Chinese; (5) outcome: (i) primary outcome measures: clinical efficacy, according to response evaluation criteria in solid tumors (RECIST) [19]; complete response: after treatment, the tumor was completely absorbed and lasted for at least 4 weeks, without new lesions; partial response: after treatment, the product of the two diameters of the tumor decreased by at least 1/2, and no new lesions appeared; stable disease: after treatment, the two-diameter product of the tumor decreased by up to 1/2, or increased by up to 1/4, lasting for at least 4 weeks, and no new lesions appeared; progressive disease: after treatment, the product of the two diameters of the tumor increased by at least 1/4 or new lesions appeared. Clinical response rate (%) =  (complete response patients + partial response patients)/all subjects × 100%. (ii) Secondary outcomes: fatigue status, patients were evaluated with the Piper Fatigue Scale (PFS) [20]. For binary variable data, the improvement was based on the proportion of patients with mild fatigue to all patients and quality of life measured by the Karnofsky Performance Status (KPS) [21]. (6) Adverse drug reactions or adverse events were evaluated by the classification standard of common adverse reactions of anticancer drugs (WHO).

### 2.3. Exclusion Criteria

The exclusion criteria were as follows: (1) literature without any of the outcomes in the inclusion criteria; (2) literature with random sequence errors; (3) literature without complete outcome data; and (4) duplicate literature and irrelevant literature.

### 2.4. Quality Assessment and Data Extraction

Two investigators independently screened the articles. First, we eliminated duplicate literature. Second, we excluded the literature that did not meet the inclusion criteria by reviewing the titles and abstracts. Third, we rescreened the full text of the literature that may meet the inclusion criteria to determine whether it was finally included or not and cross-checked them. In case of disagreement, discuss and resolve it with a third investigator. The two investigators independently extracted the data and cross-checked them. The study characteristics extracted were author, published year, sample size, tumor type, sex, age, intervention measures, course of treatment, outcome, etc. In case of disagreement, discuss and resolve it with a third investigator.

The RCT bias risk assessment tool recommended by the Cochrane manual was used to assess the bias risk of the study, including (1) generation of random sequences, (2) allocation concealment, (3) blind method of subjects and researchers, (4) blind method of outcome evaluators, (5) completeness of outcome data, (6) selective reporting, and (7) other biases. Evaluation results were classified as “low bias risk,” “high bias risk,” and “unclear bias risk”.

### 2.5. Data Synthesis and Statistical Analysis

We conducted the meta-analysis by RevMan5.3 software and Stata 14.0 software (STATA Corporation, College Station, TX). For continuous variables, if the measuring tools were the same, weighted mean difference (WMD) was used. When the same variable was measured by different tools, we used standardized mean difference (SMD) and its 95% confidence interval (95% CI) as the effect analysis statistic. For binary variables, the relative risk degree (RR) and its 95% confidence interval (95% CI) were used as the effect analysis statistics. We tested the heterogeneity of the included studies by the *χ*^2^ test and judged it according to *I*^*2*^. If *P* > 0.10 and *I*^*2*^ < 50%, it indicated that there was no statistical heterogeneity among the studies, and the fixed-effect model was adopted for the combined analysis. If not, the random-effect model was used. Meanwhile, in order to deal with the heterogeneity between studies, the subgroup analysis was carried out to explore the causes. We used Begg's funnel plot and Egger's regression analysis to test publication bias and sensitivity analysis to test the stability of the results. A *P* value < 0.05 was considered to suggest statistical heterogeneity.

## 3. Results

**Figure 1:**
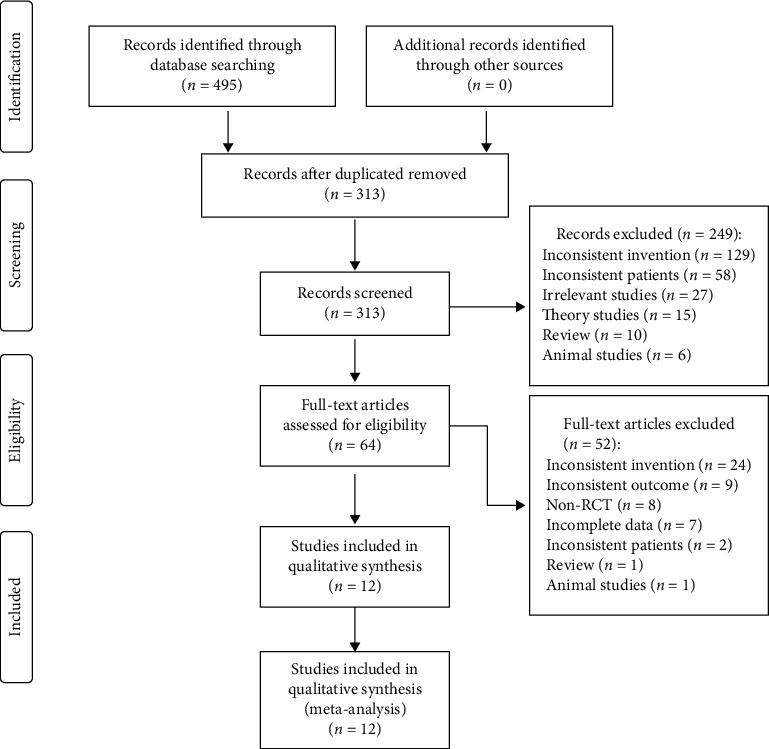
Flowchart of study identification and selection.

495 articles were retrieved through database search, and 313 articles were obtained after removing duplicates. After reading titles and abstracts, 249 articles were removed. 52 articles were excluded by further reading the full text, and 12 articles [22–33] were finally included. The literature screening process and results are shown in Figure 1.

### 3.1. The Basic Characteristics of the Inclusion Study

There were 1005 participants in 12 articles [22–33]. The subjects were divided into lung cancer, gastric cancer, and mixed cancer. TCMJ included compound Kushen injection, Aidi injection, Kangai injection, Shenmai injection, and Shenqi Fuzheng injection; the efficacy of TCMJ was mainly divided into heat-clearing effect and tonifying category. The course of treatment was 2–8 weeks. 9 studies [24–30, 32, 33] used the Piper Fatigue Scale to evaluate the fatigue status of CRF patients; 4 studies [23, 25, 29,31] used the Karnofsky Performance Status score to evaluate the quality of life of CRF patients; 6 studies [22–24, 27, 31, 32] evaluated the clinical efficacy of CRF patients according to response evaluation criteria in solid tumors; four studies [22, 24–26] evaluated the drug safety of patients with CRF using the classification standard of common adverse reactions of anticancer drugs (WHO). The basic characteristics of the included studies are shown in Table 1; the intervention measures are shown in Table 2; the outcome data are shown in Table 3.

### 3.2. Results of Risk Assessment of Bias in Included Studies

**Figure 2:**
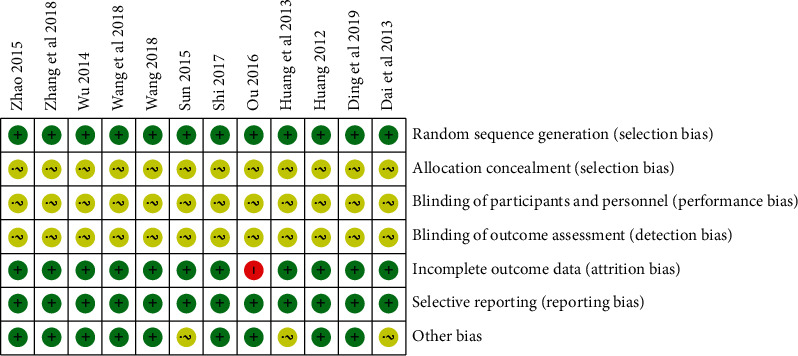
Assessment of risk of bias of included studies.

According to the Cochrane bias risk assessment tool, the quality of 12 articles [22–33] was evaluated. All of them were randomized controlled trials. All the articles [22–33] did not mention the allocation concealment and the blind method, so they were evaluated as unclear bias risk. One article [26] was evaluated as high bias risk because of incomplete data. None of the articles [22–33] reported results selectively. Three articles [22, 25, 27] had unknown baseline conditions, so the risk of bias was assessed as unclear (Figure 2).

### 3.3. Results of Meta-Analysis

#### 3.3.1. Clinical Efficacy

Six studies [22–24, 27, 31, 32] evaluated the clinical efficacy of patients with CRF according to RECIST, and there was no statistical heterogeneity among the results of each study (*P*=0.45, *I*^*2*^ = 0%). Therefore, we used a fixed-effect model for combined analysis. The results showed that the TCMJ group was superior to the control group in improving the clinical efficacy of CRF patients (RR = 1.24, 95% CI: 1.05–1.46, *P*=0.01).

**Figure 3:**
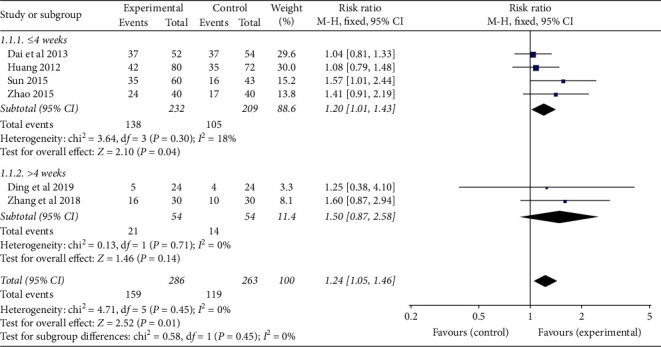
A meta-analysis of the clinical efficacy of traditional Chinese medicine injection on CRF patients.

According to the course of treatment, they were divided into two subgroups (≤4 weeks and >4 weeks) to analyze the clinical efficacy of CRF patients. ① ≤4 weeks: four studies were included, and there was no statistical heterogeneity among the results of each study (*P*=0.30, *I*^*2*^ = 18%). The results showed that the TCMJ group was better than the control group in improving the clinical efficacy of CRF patients (RR = 1.20, 95% CI: 1.01–1.43, *P*=0.04). ② >4 weeks: two studies were included, and there was no statistical heterogeneity among the results of each study (*P*=0.71, *I*^*2*^ = 0%). The results showed that the clinical efficacy of the TCMJ group was similar to that of the control group in improving the clinical efficacy of CRF patients (RR = 1.50, 95% CI: 0.87–2.58, *P*=0.14). (Figure 3)

**Figure 4:**
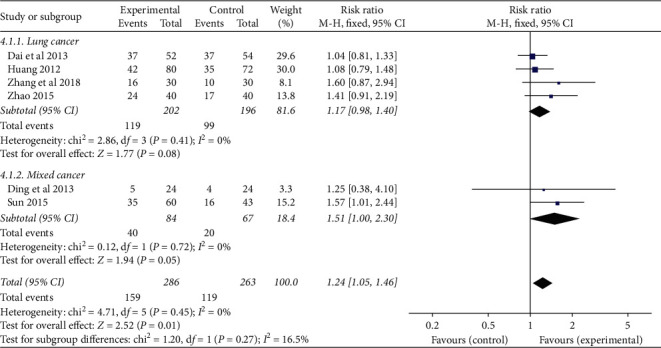
A meta-analysis of the clinical efficacy of traditional Chinese medicine injection on CRF patients.

According to the type of cancer, they were divided into two subgroups (lung cancer group and mixed cancer group) to analyze the clinical efficacy of CRF patients. ① Lung cancer: four studies evaluated the clinical efficacy of CRF in lung cancer patients, and there was no statistical heterogeneity among the results (*P*=0.41, *I*^*2*^ = 0%). The results showed that the TCMJ group was comparable to the control group in improving the clinical efficacy of CRF in lung cancer patients (RR = 1.17, 95% CI: 0.98–1.40, *P*=0.08). ② Mixed cancer: two studies evaluated the clinical efficacy of CRF in mixed cancer patients, and there was no statistical heterogeneity among the results (*P*=0.72, *I*^*2*^ = 0%). The results showed that the TCMJ group was comparable to the control group in improving the clinical efficacy of CRF in mixed cancer patients (RR = 1.51, 95% CI: 1.00–2.30, *P*=0.05) (Figure 4).

**Figure 5:**
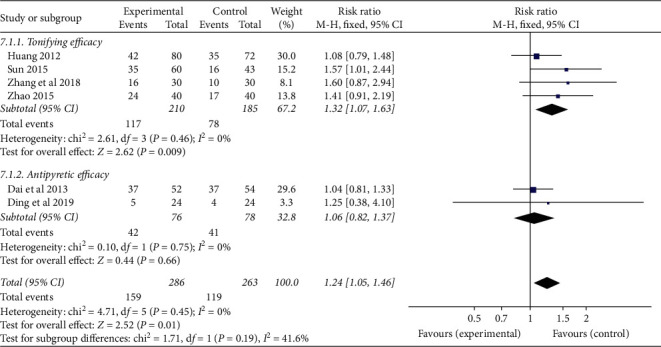
A meta-analysis of the clinical efficacy of traditional Chinese medicine injection on CRF patients.

According to the efficacy of traditional Chinese medicine injection, they were divided into two subgroups (tonifying efficacy group and heat-clearing efficacy group) to analyze the clinical efficacy of CRF patients. ① Tonifying efficacy group: four studies used tonifying efficacy TCMJ to evaluate the clinical efficacy of CRF patients, and there was no statistical heterogeneity among the results of each study (*P*=0.46, *I*^*2*^ = 0%). The results showed that the tonifying efficacy TCMJ group had a better effect than the control group (RR = 1.32, 95% CI: 1.07–1.63, *P*=0.009). ② Heat-clearing efficacy group: in two studies, heat-clearing TCMJ was used to evaluate the clinical efficacy of CRF patients, and there was no statistical heterogeneity between the results of each study (*P*=0.75, *I*^*2*^ = 0%). The results showed that the heat-clearing TCMJ group was similar to the control group in improving the clinical efficacy of CRF patients (RR = 1.06, 95% CI: 0.82–1.37, *P*=0.66) (Figure 5).

#### 3.3.2. Fatigue Status

*(1) Binary Variable Data*. In 8 studies [24–30, 32], the Piper Fatigue Scale was used to evaluate the fatigue status of CRF patients, and the results were described by binary variables. There was no statistical heterogeneity among the results of each study (*P*=0.11, *I*^*2*^ = 40%), so the fixed-effect model was used for combined analysis. The results showed that the TCMJ group was superior to the control group in improving the fatigue status of CRF patients (RR = 1.44, 95% CI: 1.27–1.65, *P* < 0.00001).

**Figure 6:**
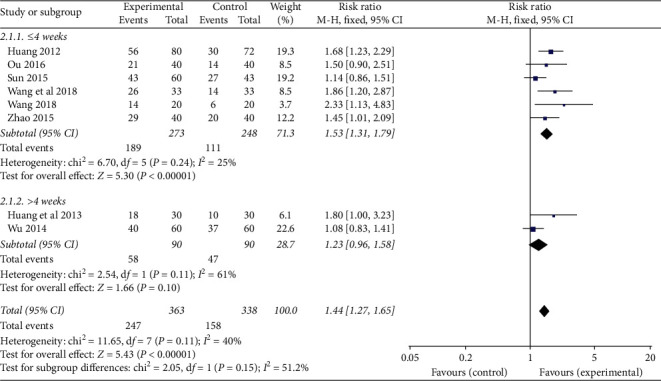
A meta-analysis of the effect of traditional Chinese medicine injection on the fatigue of CRF patients.

According to the course of treatment, the patients were divided into two subgroups (≤4 weeks and >4 weeks) to analyze the fatigue status of CRF patients. ① ≤4 weeks: 6 studies were included, and there was no statistical heterogeneity among the results of each study (*P*=0.24, *I*^*2*^ = 25%). The results showed that the TCMJ group was better than the control group in improving the fatigue of CRF patients (RR = 1.53, 95% CI: 1.31–1.79, *P* < 0.00001). ② >4 weeks: two studies were included, and there was statistical heterogeneity among the results of each study (*P*=0.11, *I*^*2*^ = 61%). The results showed that the TCMJ group was similar to the control group in improving the fatigue status of CRF patients (RR = 1.23, 95% CI: 0.96–1.58, *P*=0.10) (Figure 6).

**Figure 7:**
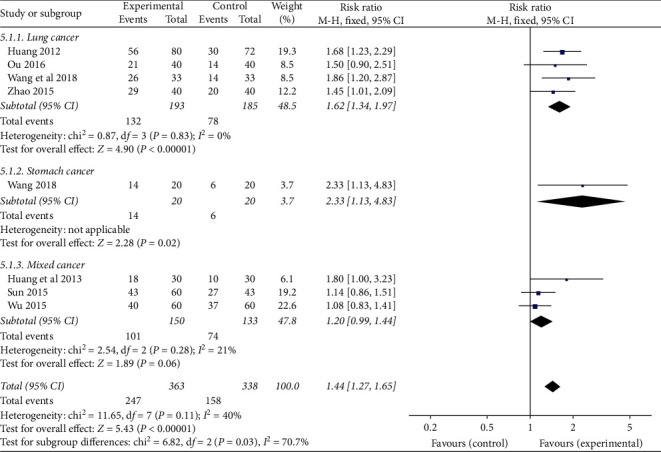
A meta-analysis of the effect of traditional Chinese medicine injection on the fatigue of CRF patients.

Three subgroups (lung cancer group, gastric cancer group, and mixed cancer group) were divided by cancer type to analyze the fatigue status of CRF patients. ① Lung cancer: four studies were included, and there was no statistical heterogeneity among the results of each study (*P*=0.83, *I*^*2*^ = 0%). The results showed that the TCMJ group was superior to the control group in improving the fatigue status of CRF patients with lung cancer (RR = 1.62, 95% CI: 1.34–1.97, *P* < 0.00001). ② Gastric cancer: one study evaluated fatigue status in gastric cancer patients with CRF. Descriptive analysis result showed that the TCMJ group had a better effect than the control group (RR = 2.33, 95% CI: 1.13–4.83, *P*=0.02). ③ Mixed cancer: three studies evaluated the fatigue status of CRF in patients with mixed cancer, and there was no statistical heterogeneity among the results of each study (*P*=0.28, *I*^*2*^ = 21%). The results showed that the TCMJ group was comparable to the control group in improving the fatigue of CRF in patients with mixed cancer (RR = 1.20, 95% CI: 0.99–1.44, *P*=0.06) (Figure 7).

**Figure 8:**
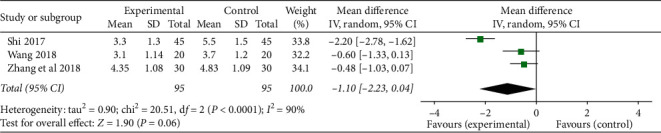
A meta-analysis of the effect of traditional Chinese medicine injection on the fatigue of CRF patients.

*(2) Continuous Variable Data*. Three studies [29, 31, 33] evaluated fatigue status of CRF patients with the Piper Fatigue Scale and described the results with mean ± standard deviation before and after treatment. The results showed that there was heterogeneity among the results of each study (*P* < 0.0001, *I*^*2*^ = 90%), and the TCMJ group was equivalent to the control group in improving the fatigue status of CRF patients (MD = −1.10, 95% CI: −2.23–0.04, *P*=0.06) (Figure 8). However, after excluding one study [33], there was no statistical heterogeneity among the results (*P*=0.80, *I*^*2*^ = 0%), and it was found that its heterogeneity may be related to the course of treatment (2 weeks in [33], while more than 2 weeks in [29] and [31]). Furthermore, we used the fixed-effect model for combined analysis. The results showed that TCMJ was superior to the control group in improving the fatigue status of CRF patients (MD = −0.52, 95% CI: −0.96–0.09, *P*=0.02).

#### 3.3.3. Quality of Life

 Four studies [23, 25, 29, 31] used the Karnofsky Performance Status score to evaluate the quality of life of CRF patients, and there was statistical heterogeneity among the results of each study (*P*=0.02, *I*^*2*^ = 70%), so the random-effect model was used for combined analysis. The results showed that the TCMJ group was superior to the control group in improving the quality of life of CRF patients (MD = 8.34, 95% CI: 3.31–13.37, *P*=0.001).

**Figure 9:**
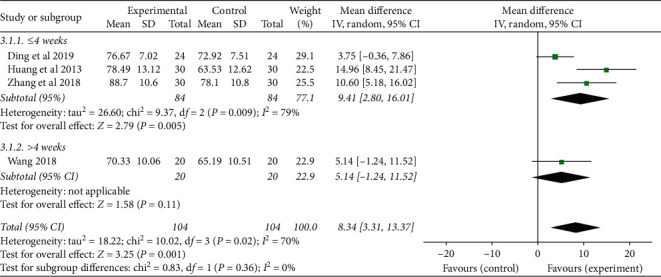
A meta-analysis of the effect of traditional Chinese medicine injection on the quality of life of CRF patients.

According to the course of treatment, the patients were divided into two subgroups (≤4 weeks and >4 weeks) to analyze the quality of life of CRF patients. ① ≤4 weeks: three studies were included, and there was statistical heterogeneity among the results of each study (*P*=0.009, *I*^*2*^ = 79%). The results showed that the TCMJ group was better than the control group in improving the quality of life of patients with CRF (MD = 9.41, 95% CI: 2.80–16.01, *P*=0.005). ② >4 weeks: one study evaluated the quality of life of patients with CRF, and descriptive analysis result showed that the TCMJ group was comparable to the control group in improving the quality of life of CRF patients (MD = 5.14, 95% CI: −1.24–11.52, *P*=0.11) (Figure 9).

**Figure 10:**
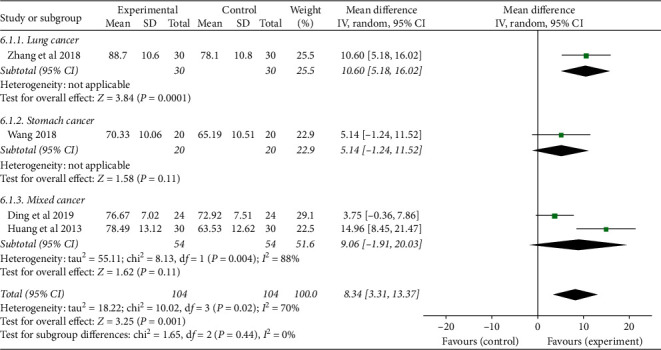
A meta-analysis of the effect of traditional Chinese medicine injection on the quality of life of CRF patients.

According to the type of cancer, the patients were divided into three subgroups (lung cancer group, gastric cancer group, and mixed cancer group) to analyze the quality of life of CRF patients. ① Lung cancer: one study evaluated the quality of life of CRF patients, and descriptive analysis result showed that the TCMJ group was better than the control group in improving the quality of life of CRF patients (MD = 10.60, 95% CI: 5.18–16.02, *P*=0.0001). ② Gastric cancer: one study evaluated the quality of life of gastric cancer patients with CRF. Descriptive analysis result showed that the TCMJ group was comparable to the control group in improving the quality of life of gastric cancer patients with CRF (MD = 5.14, 95% CI: −1.24–11.52, *P*=0.11). ③ Mixed cancer: two studies were included, and there was statistical heterogeneity among the results of each study (*P*=0.004, *I*^*2*^ = 88%). The results showed that the TCMJ group was comparable to the control group in improving the quality of life of CRF patients with mixed cancer (Figure 10).

#### 3.3.4. Safety Analysis

Four studies [22, 24–26] evaluated the drug safety of CRF patients using the classification standard of common adverse reactions of anticancer drugs (WHO), and most of the adverse reactions were myelosuppression and digestive system reactions. In most cases of adverse reactions, the number of adverse reactions in the TCMJ group was less than that in the control group, indicating that TCMJ had a good safety. The details are shown in Table 4.

#### 3.3.5. Sensitivity Analysis

**Figure 11:**
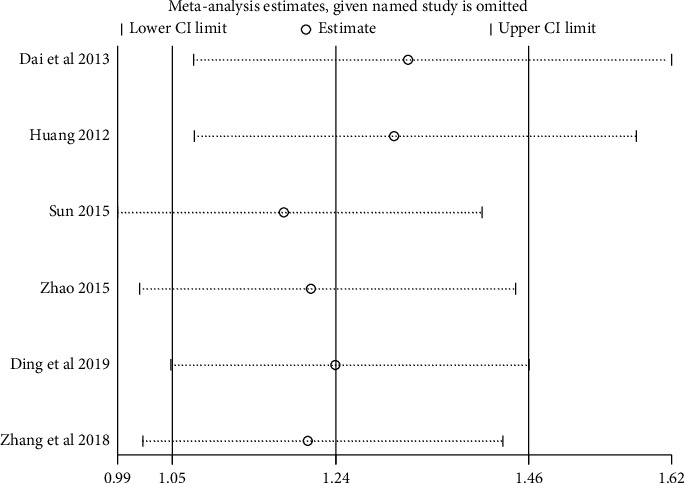
Results of sensitivity analysis of clinical efficacy.

**Figure 12:**
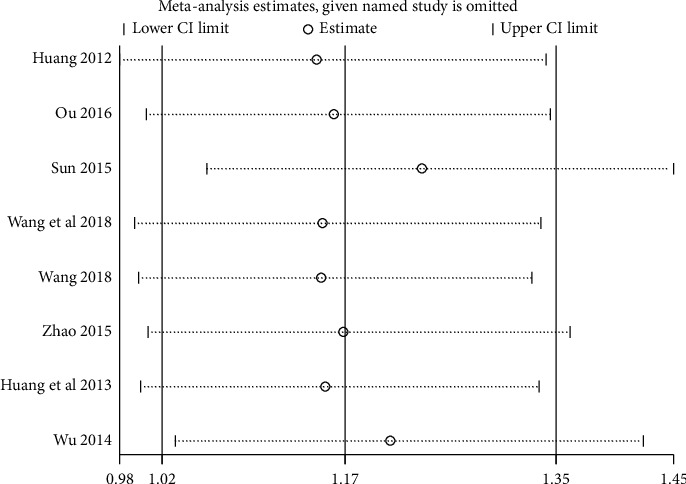
Results of sensitivity analysis of fatigue status (binary data).

**Figure 13:**
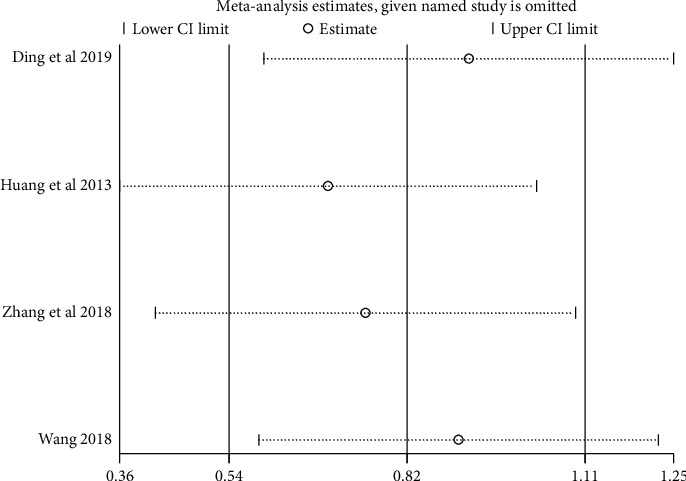
Results of quality of life sensitivity analysis.

**Figure 14:**
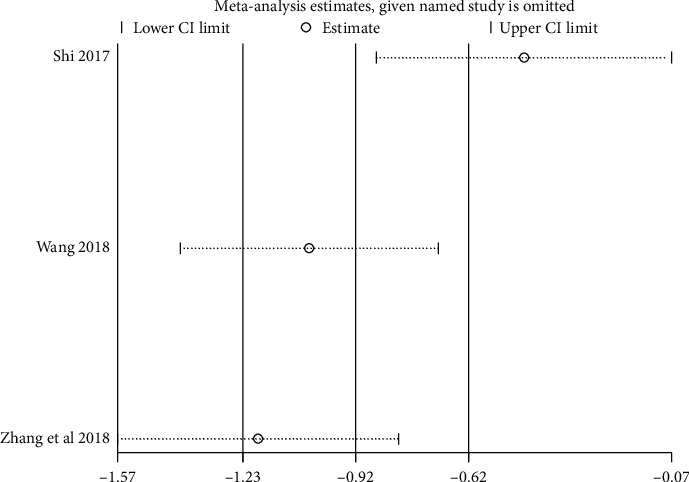
Results of sensitivity analysis of fatigue status (continuous data).

We performed sensitivity analysis to test the stability of the results, and we found that the results of each indicator were stable, as shown in Figures 11–14.

#### 3.3.6. Publication Bias

**Figure 15:**
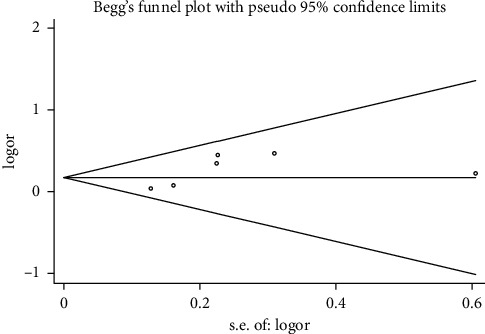
Begg's funnel chart analysis of clinical efficacy.

**Figure 16:**
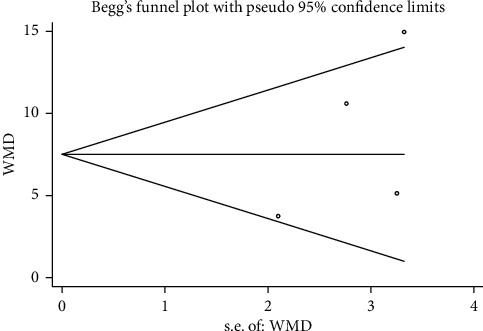
Begg's funnel chart analysis of life quality.

**Figure 17:**
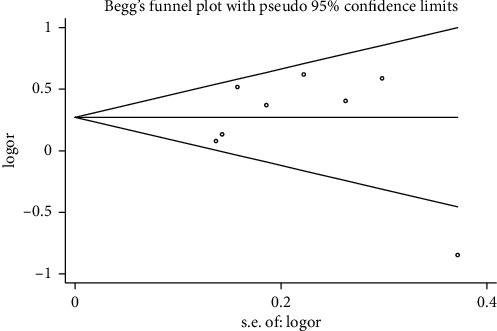
Begg's funnel chart analysis of fatigue state (binary data).

**Figure 18:**
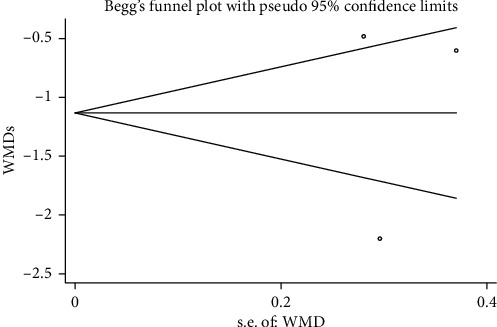
Begg's funnel chart analysis of fatigue state (continuous data).

Begg's funnel plot and Egger's test were performed to evaluate the publication bias of the included studies (Figures 15–18). The results showed no statistically significant difference: clinical efficacy (Begg's test: *P*=0.188; Egger's test: *P*=0.147); fatigue status (binary data) (Begg's test: *P*=1.000; Egger's test: *P*=0.910); fatigue status (continuous data) (Begg's test: *P*=0.602; Egger's test: *P*=0.888); and quality of life (Begg's test: *P*=0.497; Egger's test: *P*=0.200).

## 4. Discussion

CRF is one of the most common and difficult-to-treat symptoms caused by multifactor interaction, which runs through the whole process of tumorigenesis, development, treatment, and prognosis [34]. As CRF is a systemic symptom, the specific pathogenesis is not clear at present [35]. Studies have shown that the possible mechanisms of CRF include cytokine dysfunction, hypothalamus-pituitary-adrenal axis dysfunction, circadian rhythm disorders, vagal afferent nerve activation, serotonin dysregulation, muscle metabolism changes, and dysregulation of adenosine triphosphate [36, 37]. Currently, the intervention measures of CRF are divided into nondrug intervention and drug-based intervention. Nondrug intervention mainly includes physical activity, massage therapy, psychosocial intervention, sleep cognitive behavioral therapy, and bright white light therapy. Drug-based intervention is mainly based on central stimulant (methylphenidate), but its application is limited due to its status as a psychotropic controlled drug [7, 38, 39]. Therefore, looking for other potentially effective drugs is the key to CRF treatment. Traditional Chinese medicine (TCM) believes that CRF is due to the impact of cancer and chemotherapy, resulting in deficiency of qi and blood and disharmony between yin and yang. In view of this basic pathogenesis, replenishing qi and helping vital qi is often taken as the main treatment. Clinical practice has proved that traditional Chinese medicine has certain advantages in reducing the toxic and side effects of radiotherapy and chemotherapy in tumor patients, improving patients' clinical symptoms, and improving patients' immunity [40]. TCMJ is not only the product of the combination of traditional medicine theory and modern production technology but also an important product of the modernization of traditional Chinese medicine. Traditional Chinese medicine injection has the characteristics of high bioavailability, definite curative effect, and rapid action. Commonly used clinical injections of traditional Chinese medicine include Kangai injection, Aidi injection, Shenmai injection, and Shenqi Fuzheng injection. The prescription contains Radix Astragali, ginseng, *Sophora flavescens*, *Ophiopogon*, *Mylabris*, *Acanthopanax senticosus*, etc. In traditional Chinese medicine, Radix Astragali tonifies qi and nourishes blood; ginseng invigorates vitality, nourishes the spleen and lung, promotes saliva and quenches thirst, calms the mind, and promotes wisdom; *Sophora flavescens* clears heat and detoxifies the body, disperses knots, and relieves pain; ophiopogonis nourishes Yin and promotes saliva, moistens the lung, and clears the heart; *Mylabris* removes blood stasis and disperses knots; *Acanthopanax senticosus* promotes wisdom and calms the mind. According to modern pharmacological studies, the main component of Radix Astragali is Astragalus polysaccharides, which plays an immunomodulatory role by activating NK cells, B lymphocytes, and T lymphocytes and promoting the synthesis of dendritic cells and the secretion of cytokines, etc. [41]. The main active ingredient of ginseng is ginsenoside, which has the effects of antitumor, antioxidation, antiaging, and cardiotonic [42]. Matrine, the main extract of *Sophora flavescens*, kills tumor cells by affecting telomerase [43]. *Ophiopogon* has the effects of immune regulation, antiaging, antifatigue, and antitumor [44]. *Mylabris* can induce tumor cell apoptosis by affecting the nucleic acid metabolism of cancer cells [45]. *Acanthopanax senticosus* has the effects of improving sleep and antidepression [46]. Animal experiments have shown that Shenqi Fuzheng injection can increase the level of adenine nucleoside triphosphate in tired mice and improve the fatigue state of mice. Clinical studies have shown that TCMJ plays an important role in promoting the activity of the T cell group and improving the immunity of the body [47–49]. Shenmai injection can improve immune function, enhance antitumor ability, and significantly reduce the levels of plasma endotoxin and INF-*α* [31]. The clinical study by Qian Si [39] found that Aidi injection could improve the fatigue of CRF patients.

The outcome measures of the study were clinical efficacy, Piper Fatigue Scale, Karnofsky Performance Status score, and adverse reactions. The clinical efficacy was evaluated by RECIST. The Piper Fatigue Scale has been widely used to evaluate CRF patients at home and abroad. The Karnofsky performance status score is a score used to evaluate the health status and treatment tolerance of tumor patients, which has been widely used in recent years. The results of this meta-analysis showed that TCMJ could improve clinical efficacy, fatigue status, and quality of life of CRF patients, but there was no statistical significance in the fatigue score. The course of treatment was found to be the possible reason for it by sensitivity analysis, but subgroup analysis could not be performed for further verification due to the lack of studies. We used RECIST to evaluate the therapeutic effect of TCMJ on tumor and found that TCMJ could improve the treatment effect. TCMJ had antitumor effect, and its synergistic effect with chemotherapeutic drugs could improve the clinical efficacy. The improvement of fatigue status and life quality was not only related to the fact that TCMJ could enhance body immunity and reduce toxic and side effects but also related to the antitumor effect of TCMJ, which involved in the improvement of clinical efficacy. In addition, the number of cases of most adverse reactions in the TCMJ group was less than that in the chemotherapy alone group because TCMJ could reduce toxic and side effects. We performed a subgroup analysis based on the type of cancer, course of treatment, and efficacy of TCMJ. The results showed that TCMJ could improve the clinical efficacy, fatigue status, and quality of life in a short time (≤4 weeks). Chemotherapy will cause varying degrees of fatigue. Platinum-containing chemotherapeutic drugs are more likely to cause fatigue than nonplatinum chemotherapeutic drugs [36, 50]. Platinum-containing chemotherapeutic drugs, easily leading to nausea, vomiting, and other symptoms, could reduce appetite and bring more severe fatigue to patients due to lack of energy, nutrition, and positive emotional status. All the chemotherapy regimens we included contained platinum. When the course of treatment was less than 4 weeks, the toxic and side effects of chemotherapy were severe, and the fatigue symptoms of patients were obvious. Therefore, TCMJ treatment could significantly improve the fatigue. However, when the course of treatment was longer than 4 weeks, with the treatment of tumor, the impact of tumor on fatigue was reduced, and the toxic and side effects of chemotherapy were also gradually reduced. In this case, we could not observe statistical significance in improving fatigue with TCMJ. In addition, we observed that tonic TCMJ had significant curative effect, and its mechanism lay in the basic pathogenesis of deficiency of vital qi. We also found that there were significant differences in improvement of fatigue status and life quality in patients with lung cancer. The possible reason was that the lesions of patients with lung cancer were mainly in the lung, while the damage to the spleen and stomach was not serious. The fatigue status and life quality of lung cancer patients could be improved by the method of invigorating vital qi and reinforcing earth (spleen) to strengthen metal (lung). However, not only the spleen and stomach of patients with gastric cancer but also the functions of multiple viscera of patients with mixed cancer were seriously damaged, so their curative effect was not good.

## 5. Limitations

(1) The 12 included articles involved a variety of traditional Chinese medicine injections, but we only divided them into two categories according to the efficacy of TCMJ. However, the composition of traditional Chinese medicine in each injection was not the same, which may lead to heterogeneity among studies. Further research on appropriate clinical treatment methods needs to be carried out. (2) In addition to clinical efficacy, Piper Fatigue Scale, KPS score, and adverse reactions, the evaluation indexes of TCMJ for CRF also included the TCM syndrome score, biochemical index, etc., which were not analyzed due to the limitation of the original study. (3) There were some differences in the basic characteristics of the included studies, such as age, gender, sample size, and types of chemotherapy drugs, which all increased the heterogeneity of the studies. (4) None of the included literatures mentioned the implementation of the blind method, which may have a certain impact on the determination of outcome indicators, resulting in implementation bias.

## 6. Conclusions

In summary, the current research evidence showed that in a short course of treatment (≤4 weeks), tonic TCMJ had a significant effect on improving the clinical efficacy of CRF patients. TCMJ, with good safety, had advantages in improving the fatigue status of CRF patients with lung cancer and gastric cancer, as well as life quality of lung cancer patients with CRF. Limited by the sample size and quality of the included study, the results of this analysis should be treated with caution. In the future, multicenter, large-sample, and high-quality randomized controlled trials should be carried out under strict scientific research design. The standard measurement index for evaluating curative effect should be adopted to make the results more objective. Follow-up after discharge should be added to the study to evaluate the long-term therapeutic effect and adverse reactions of TCMJ on CRF.

## Figures and Tables

**Table 1:** Baseline characteristics of included studies.

Study	State	Age	Gender (male/female)	Tumor type and stage	No. of patients (start)	No. of patients (end)	Outcome
Dai et al. [22]	China	T: 38～72 C: 39～71	T: 28/24 C: 31/23	Lung cancer III∼IV	106	106	①④
Ding et al. [23]	China	T: 63.92 ± 7.05 C: 65.46 ± 8.46	T: 14/10 C: 13/11	Lung, stomach, and colorectal cancer IIIB∼IV	48	48	①②
Huang et al. [24]	China	T: 60～77 C: 60～76	T: 49/31 C: 45/27	Lung cancer III∼IV	152	152	①②④
Huang et al. [25]	China	T: 47.4 ± 3.6 C: 46.6 ± 4.3	T: 20/10 C: 22/8	Esophageal, colorectal, nasopharyngeal, lung, and breast cancer III∼IV	60	60	②③④
Ou [26]	China	T: 60.8 ± 7.2 C: 61.2 ± 7.6	T: 24/16 C: 23/17	Lung cancer II∼III	80	80	②④
Sun [27]	China	36～80	64/38	Gastric, esophageal, and colorectal cancer	103	103	①②
Wang et al. [28]	China	T: 55.4 ± 4.5 C: 57.5 ± 4.7	T: 18/15 C: 20/13	Lung cancer	66	66	②
Wang [29]	China	T: 65.9 ± 10.198 C: 64.4 ± 12.713	T: 12/8 C: 13/7	Stomach cancer	40	40	②③
Wu [30]	China	T: 58.6 ± 7.98 C: 58.1 ± 8.05	T: 32/28 C: 36/24	Lung, liver, stomach, breast, colorectal, esophageal, and nasopharyngeal cancer	120	120	②
Zhang et al. [31]	China	T: 58.5 ± 7.7 C: 59.3 ± 7.5	T: 15/15 C: 16/14	Lung cancer II∼III	60	60	①③
Zhao [32]	China	T: 57.8 ± 4.9 C: 58.2 ± 4.7	T: 25/15 C: 23/17	Lung cancer II∼IV	80	80	①②
Shi [33]	China	T: 62.5 ± 5.0 C: 62.0 ± 4.8	T: 26/19 C: 25/20	Lung cancer II∼III	90	90	②

T, treatment group; C, control group; ① clinical efficacy; ② fatigue status; ③ life quality evaluation;④ adverse reactions.

**Table 2:** Description of interventions.

Study	Interventions of treatment group			Interventions of control group	Duration (weeks)
	Chemotherapeutic drugs	Traditional Chinese medicine injection	Efficacy of TCMJ	Chemotherapeutic drugs	
Dai et al. [22]	Lung adenocarcinoma: gemcitabine 1000 mg/m2, d1, 8, cisplatin75 mg/m2, d1, 4 Lung squamous cell carcinoma: docetaxel75 mg/m2,d1, cisplatin75 mg/m2,d1, 4	Compound Kushen injection 20 ml, qd (*Sophora flavescens*, Baituling)	Clearing heat and promoting dampness, cooling blood and detoxifying, and dispelling knots and relieving pain	Lung adenocarcinoma: gemcitabine 1000 mg/m2,d1, 8+, cisplatin 75 mg/m2,d1, 4 Lung squamous cell carcinoma: docetaxel 75 mg/m2,d1+, cisplatin 75 mg/m2,d1, 4	2
Ding et al. [23]	NSCLC: pemetrexed, carboplatin/nedaplatin Gastric cancer: capecitabine, oxaliplatin Colorectal cancer: capecitabine, oxaliplatin	Aidi injection 80 ml, qd (Radix Astragali, ginseng, *Acanthopanax senticosus, Mylabris*)	Clearing heat and detoxifying, and eliminating blood stasis and dispersing knots	NSCLC: pemetrexed + carboplatin/nedaplatin Gastric cancer: capecitabine + oxaliplatin Colorectal cancer: capecitabine + oxaliplatin	8
Huang et al. [24]	Lung cancer: vinorelbine 25 mg/m2,d1, 8, carboplatin 300 mg/m2,d1	Kangai injection 60 ml, qd (ginseng, Radix Astragali, *Sophora flavescens*)	Replenish qi and help vital qi	Lung cancer: vinorelbine 25 mg/m2,d1, 8+ carboplatin 300 mg/m2,d1	2
Huang et al. [25]	Esophageal cancer: cisplatin, 5-fluorouracil, calcium folinate Colorectal cancer: oxaliplatin, 5-fluorouracil, calcium folinate Nasopharyngeal carcinoma: cisplatin, 5-fluorouracil Lung cancer: gemcitabine, cisplatin Breast cancer: cyclophosphamide, pirarubicin, 5-fluorouracil or cisplatin, pirarubicin	Shenmai injection 40 ml, qd (ginseng, *Ophiopogon japonicus*)	Replenish qi, nourish yin, and nourish fluid	Esophageal cancer: cisplatin, 5-fluorouracil, calcium folinate Colorectal cancer: oxaliplatin, 5-fluorouracil, calcium folinate Nasopharyngeal carcinoma: cisplatin, 5-fluorouracil Lung cancer: gemcitabine, cisplatin Breast cancer: cyclophosphamide, pirarubicin, 5-fluorouracil or cisplatin, pirarubicin	6
Ou [26]	Lung cancer: gemcitabine 1000 mg/m2,d1, 8, cisplatin 75 mg/m2,d1-3	Kangai injection 40 ml, qd (ginseng, Radix Astragali, *Sophora flavescens*)	Replenish qi and help vital qi	Lung cancer: gemcitabine 1000 mg/m2,d1, 8, cisplatin 75 mg/m2,d1-3	2
Sun [27]	NA	Kangai injection (ginseng, Radix Astragali, *Sophora flavescens*)	Replenish qi and help vital qi	NA	4
Wang et al. [28]	NSCLC: paclitaxel, carboplatin; gemcitabine, carboplatin; pemetrexed, cisplatin	Kangai injection 50 ml, qd (ginseng, Radix Astragali, *Sophora flavescens*)	Replenish qi and help vital qi	NSCLC: paclitaxel, carboplatin; gemcitabine, carboplatin; pemetrexed, cisplatin	2
Wang [29]	Gastric cancer: oxaliplatin 130 mg/m2,d1 + tegafur capsule 40∼60 mg	Shenqi Fuzheng injection 250 ml, qd (*Codonopsis pilosula*, Radix Astragali)	Replenish qi and help vital qi	Gastric cancer: oxaliplatin 130 mg/m2,d1 + tegafur capsule 40∼60 mg	3
Wu [30]	Lung cancer: gemcitabine, cisplatin Liver cancer: daunorubicin, 5-fluorouracil, cisplatin Gastric cancer: calcium folinate, fluorouracil, etoposide Breast cancer: cyclophosphamide, pirarubicin, 5-fluorouracil Colorectal cancer: oxaliplatin, 5-fluorouracil, calcium folinate Esophageal cancer: cisplatin, 5-fluorouracil, calcium folinate Nasopharyngeal carcinoma: cisplatin, 5-fluorouracil	Shenmai injection 60 ml, qd (ginseng, *Ophiopogon japonicus*)	Replenish qi, nourish yin, and nourish fluid	Lung cancer: gemcitabine, cisplatin Liver cancer: daunorubicin, 5-fluorouracil, cisplatin Gastric cancer: calcium folinate, fluorouracil, etoposide Breast cancer: cyclophosphamide, pirarubicin, 5-fluorouracil Colorectal cancer: oxaliplatin, 5-fluorouracil, calcium folinate Esophageal cancer: cisplatin, 5-fluorouracil, calcium folinate Nasopharyngeal carcinoma: cisplatin, 5-fluorouracil	8
Zhang et al. [31]	Lung cancer: gemcitabine 1000 mg/m2,d1, 8, cisplatin 25 mg/m2,d1-3	Shenmai injection 100 ml, qd (ginseng, *Ophiopogon japonicus*)	Replenish qi, nourish yin, and nourish fluid	Lung cancer: gemcitabine 1000 mg/m2,d1, 8, cisplatin 25 mg/m2,d1-3	8
Zhao [32]	Lung cancer: vinorelbine 25∼30 mg/m2, d1, 8, cisplatin 75 mg/m2,d1	Kangai injection 50 ml (ginseng, Radix Astragali, *Sophora flavescens*)	Replenish qi, nourish yin, and nourish fluid	Lung cancer: vinorelbine 25–30 mg/m2,d1, 8, cisplatin 75 mg/m2,d1	2
Shi [33]	Lung cancer: gemcitabine 1000 mg/m2,d1, 8, 15, cisplatin 30 mg/m2,d1-3	Kangai injection 40 ml, qd (ginseng, Radix Astragali, *Sophora flavescens*)	Replenish qi, nourish yin, and nourish fluid	Lung cancer: gemcitabine 1000 mg/m2,d1, 8, 15, cisplatin 30 mg/m2,d1-3	2

Note: NA, not available.

**Table 3:** Outcome data summary.

Outcome	Study	Treatment group				Control group			
		No. of patients	Baseline	No. of patients	Posttreatment	No. of patients	Baseline	No. of patients	Posttreatment
Fatigue status (binary variable data)	Wu [30]	60	27/60^#^	60	40/60^#^	60	27/60^#^	60	37/60^#^
	Sun [27]	60	16/60^#^	60	43/60^#^	43	18/43^#^	43	27/43^#^
	Ou [26]	40	11/40^#^	40	21/40^#^	40	12/40^#^	40	14/40^#^
	Wang [29]	20	1/20^#^	20	6/20^#^	20	2/20^#^	20	14/20^#^
	Wang et al. [28]	33	9/33^#^	33	26/33^#^	33	7/33^#^	33	14/33^#^
	Zhao [32]	40	13/40^#^	40	29/40^#^	40	12/40^#^	40	20/40^#^
	Huang et al. [25]	80	30/80^#^	80	56/80^#^	72	28/72^#^	72	30/72^#^
	Huang et al. [24]	30	17/30^#^	30	18/30^#^	30	15/30^#^	30	10/30^#^
Fatigue status (continuous variable data)	Wang [29]	20	4.40 ± 1.19	20	3.1 ± 1.14	20	4.37 ± 1.04	20	3.70 ± 1.20
	Zhang et al. [31]	30	5.35 ± 1.22	30	4.35 ± 1.08	30	5.38 ± 1.19	30	4.83 ± 1.09
	Shi [33]	45	4.8 ± 1.8	45	3.3 ± 1.3	45	4.6 ± 1.5	45	5.5 ± 1.5
Life quality evaluation	Ding et al. [23]	24	80.42 ± 8.59	24	76.67 ± 7.02	24	81.25 ± 7.97	24	72.92 ± 7.51
	Zhang et al. [31]	30	69.4 ± 10.6	30	88.7 ± 10.6	30	69.9 ± 10.7	30	78.1 ± 10.8
	Wang [29]	20	79.64 ± 12.98	20	70.33 ± 10.06	20	78.32 ± 12.65	20	65.19 ± 10.51
	Huang et al. [24]	30	62.46 ± 12.36	30	78.49 ± 13.12	30	63.08 ± 11.41	30	63.53 ± 12.62

Note: #: patients with mild fatigue/all patients.

**Table 4:** Summary of adverse reactions.

Study	Treatment group (cases)							Control group (cases)						
	Myelosuppression	Digestive system reaction	Fever	Muscle soreness	Allergic reaction	Neurotoxicity	Others	Myelosuppression	Digestive system reaction	Fever	Muscle soreness	Allergic reaction	Neurotoxicity	Others
Dai et al. [22]	13	16	5	21	9	12	0	21	27	16	28	14	18	0
Ding et al. [23]	Leukopenia (68)/thrombocytopenia (16)/ hemoglobin decrease (15)	48	1	0	0	12	Phlebitis (5)/abnormal liver function (4)/renal dysfunction (1)/abnormal ECG (2)/hair loss (70)	Leukopenia (61)/thrombocytopenia (15)/hemoglobin decrease (18)	48	1	0	0	14	Phlebitis (7)/abnormal liver function (9)/renal dysfunction (5)/abnormal ECG (4)/hair loss (78)
Huang et al. [24]	6	10	0	0	0	0	0	18	20	0	0	0	4	0
Ou [26]	7	13	8	15	10	10	0	17	19	10	20	7	13	0
